# Vision First? The Development of Primary Visual Cortical Networks Is More Rapid Than the Development of Primary Motor Networks in Humans

**DOI:** 10.1371/journal.pone.0025572

**Published:** 2011-09-30

**Authors:** Patricia Gervan, Andrea Berencsi, Ilona Kovacs

**Affiliations:** 1 Department of Cognitive Science, Faculty of Sciences, Budapest University of Technology and Economics, Budapest, Hungary; 2 Hungarian National Academy - Budapest University of Technology and Economics Cognitive Science Research Group, Budapest, Hungary; Istituto di Neuroscienze, Italy

## Abstract

The development of cortical functions and the capacity of the mature brain to learn are largely determined by the establishment and maintenance of neocortical networks. Here we address the human development of long-range connectivity in primary visual and motor cortices, using well-established behavioral measures - a Contour Integration test and a Finger-tapping task - that have been shown to be related to these specific primary areas, and the long-range neural connectivity within those. Possible confounding factors, such as different task requirements (complexity, cognitive load) are eliminated by using these tasks in a learning paradigm. We find that there is a temporal lag between the developmental timing of primary sensory vs. motor areas with an advantage of visual development; we also confirm that human development is very slow in both cases, and that there is a retained capacity for practice induced plastic changes in adults. This pattern of results seems to point to human-specific development of the “canonical circuits” of primary sensory and motor cortices, probably reflecting the ecological requirements of human life.

## Introduction

**Figure 1:**
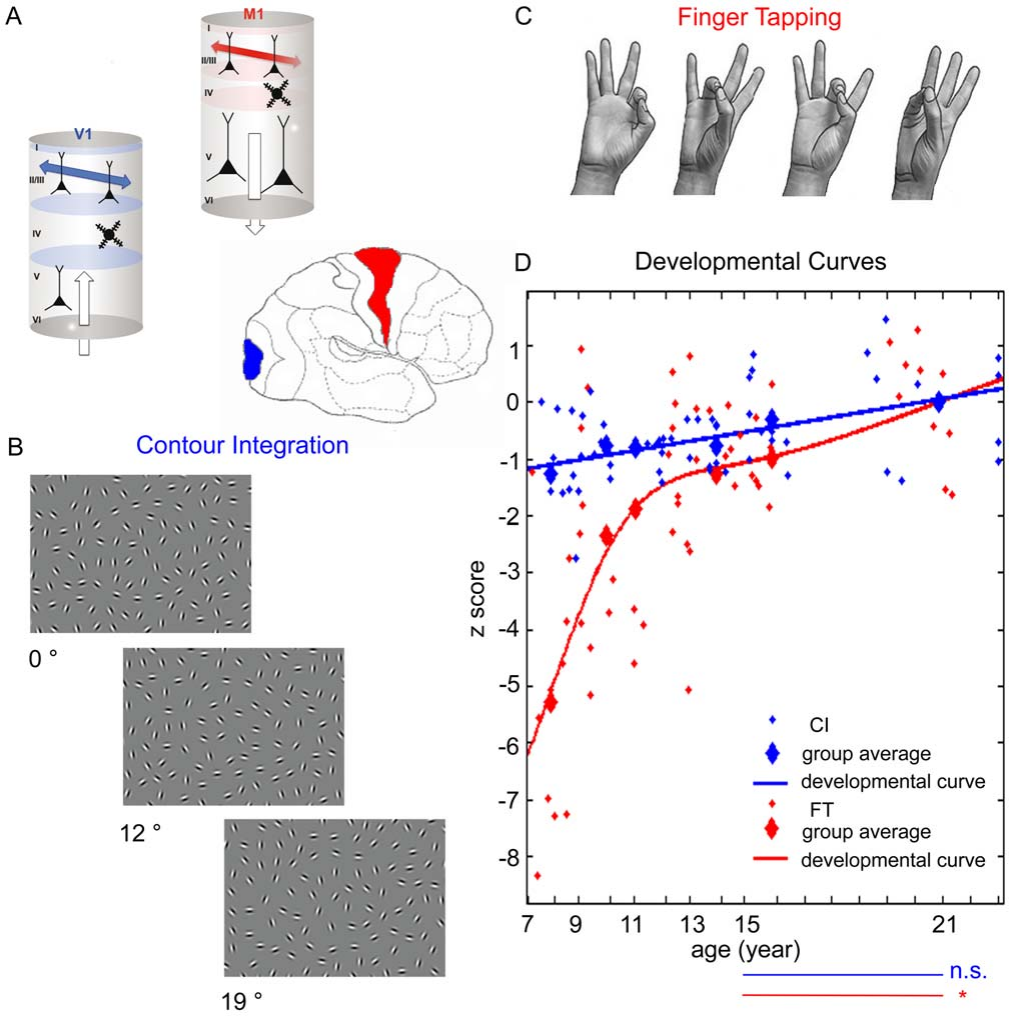
Summary of the methods and results. (A) Sideview of the human brain with the primary visual cortex (V1, Br 17) in blue, and the primary motor cortex (M1 or Br 4) in red. The cerebral cortex is generally divided into six functionally distinct layers, and the principal source of long-range lateral intralaminar connections is layer II and III, as shown in the insets corresponding to V1 and M1. (B) Contour Integration (CI) stimuli, addressing long-range connections in the primary visual cortex. The collinear chain of oriented elements forming a horizontally placed egg-shape is hidden in the background of randomly positioned and oriented elements. The panels show three levels of difficulty in the CI task. Practice and development leads to improved performance. (C) Movement-sequence in the Finger-tapping (FT) task addressing long-range connectivity of the primary motor cortex. Accuracy and speed of carrying out this sequence improves following practice and during the course of development. (D) Developmental curves in CI (blue) and in FT (red). Day 2 performance of each age-group was normalized to that of the adult performance in each task. Small symbols: individual data; large symbols: age-group average. Curve fitting was done on the age-group average values. The horizontal lines at the bottom connect two age-groups (15 and 21 y), and significance levels of the difference in performance in the two tasks, respectively, are denoted.

The development of cortical functions and the capacity of the mature brain to learn are largely determined by the establishment and maintenance of neocortical networks. The specification of long-range connectivity within larger inter-areal and more local intra-areal networks is a basic architectural requirement of cortical processing. Long-range lateral intralaminar connections between pyramidal cells (Figure 1A) seem to be a ubiquitous feature of the superficial cortical layers in, e.g., cats [1]–[3]; tree shrews [4]; and monkeys [4]–[5]. It has been suggested that these long axonal projections shape the neocortex into “canonical circuits” serving spatiotemporal integration within the functional maps [6]–[7]. The specificity of long-range connections has been extensively studied in primary sensory and motor cortices of different mammalian species. With respect to the primary visual cortex (V1 or Brodmann area 17, see Figure 1A), it has been shown that clusters of layer II/III long-range horizontal connections connect neuronal columns with similar orientation specificity in cats and monkeys [8]–[9], assumedly mediating object-related processing and visual perceptual learning in humans as well [10]–[11].

With respect to the primary motor cortex (M1; Brodmann area 4, see Figure 1A), pyramidal cells with same or similar output properties are accumulated in columns, forming elementary movement representations [12]–[14]. Collaterals of the pyramidal cells in layer II/III project horizontally as far as 3 mm long and terminate in columns with similar output to that of the original column [5]. These intrinsic connections are thought to be important in the selection and coordination of different movement representations [13], [15], in the control of different muscles around a given joint [16]–[17], or neighboring joints of the same extremity [18]. It has been proposed that the intrinsic long-range connections also mediate motor map plasticity and the learning of new motor skills in rats [19]–[21], cats [22] and primates [23].

Rough clusters of horizontal connections in V1 are present in cats and ferrets before eye opening, become refined soon thereafter [24]–[25], and the adult pattern of connections is there at birth in primates [26]. With respect to movement representation in M1, it seems to develop after the somatosensory representations and corticospinal terminations develop mature topography in cats [22], however, information is lacking with respect to the postnatal development of horizontal connectivity.

Is it a possible scenario that these “canonical circuits,” mediating basic perceptual and motor function and learning, develop similarly in different mammals, including humans? Or, alternatively, based on the obviously increased demand for human learning capacity, shall we assume that this type of long-range cortical connectivity has a human-specific developmental trend? The development of horizontal connections in layer II/III of the primary visual cortex of humans has been indicated to extend into childhood [27], corresponding to behavioral findings on the late maturation of V1-related contour integration abilities, improving until the teenage years [28]–[29]. Although little is known about the characteristics of the M1 motor representation in infants and young children, there are studies investigating the postnatal development of motor responses induced by transcranial magnetic stimulation (TMS). Motor-evoked potentials produced by TMS occur only at maximal currents in 2-year-old humans, and stimulation thresholds decrease until the age of 15 [30]–[31]. These suggest a protracted development of both sensory and motor long-range intra-areal connectivity, with the possibility of M1 ‘wiring’ taking a longer time than V1 ‘wiring.’ However, to tackle the functional development of long-range lateral intralaminar connections in humans is an intricate issue, considering the necessity to apply non-invasive measurements, and the fact that even the finest brain imaging techniques are orders of magnitude below the spatial resolution needed for such estimations.

Here we address the human development of long-range connectivity in primary visual and motor cortices, using well-established behavioral measures that have been shown to be related to these specific primary areas, and the long-range neural connectivity within those. The visual paradigm is a Contour Integration task (CI, see Figure 1B), and the motor paradigm is a sequential finger-tapping task (FT, see Figure 1C). CI has originally been developed to test the spatial integration properties of neurons with conjoint orientation preference in the primary visual cortex [32]–[33]. The presence of global, shape-dependent contextual processes at this early cortical level has been demonstrated [32], [34]–[36], indicating that long-range connectivity might contribute to object related processing, and that even primary visual processing is well beyond local feature analysis. Neural correlates, involving the correspondence between neuronal and behavioral responses in monkeys [35], direct architectural data in monkeys [9], optical imaging of contextual interactions in monkeys [37], human neuropsychology [38] and human fMRI [39]–[40] indicate the relevance of low-level visual areas integrating the contour-in-noise stimulus. Based on these studies, the possible candidate for assembling local orientation information in CI is the plexus of long-range horizontal connections in V1. The well-defined nature of stimulus processing in CI guarantees that it is a good tool to probe the development of long-range neural interactions in V1. FT is a motor coordination paradigm, where participants touch the thumb with the other fingers in a given order as quickly and precisely as possible. Combined with imaging and electrophysiological techniques, it has been an important tool to study motor learning in the last two decades. Training in FT leads to experience specific changes in M1, revealed by fMRI [41]–[42], TMS [43] and electrophysiology [44] in humans. M1 subregions contain multiple overlapping motor representations that are functionally connected through an extensive horizontal network [16]–[17], [45]–[46]. Suggested mechanisms for functional reorganization involve activity-driven synaptic strength changes in these networks [45], [47]. It is important to mention that FT performance is affected by conduction velocity of the corticospinal tract due to myelination (see the Results section). To eliminate the effect of age-related corticospinal tract conduction velocity changes, we measured maximum finger tapping speed and subtracted it from the FT data. This procedure ensured that the corrected results reflect cortical plasticity.

In addition to finding the suitable behavioral paradigms to establish maturational trajectories, comparison between the two domains requires particular consideration. Even in well-established behavioral tasks (such as CI and FT) clearly addressing long-range connectivity within primary visual and motor areas, performance might depend on a number of factors that are irrelevant in terms of the comparison of developmental rates across the two modalities. It would be precarious to directly contrast performance of different age-groups in CI and FT as there might be differences in terms of task difficulty and a potentially different impact of both subcortical mechanisms and higher level cognitive processes across modalities and across different age-groups. In order to deal with latent confounding factors we relied on a training-based design in both tasks. All observers practiced over the course of five days, allowing us to establish learning curves for each studied age-group. It has been indicated that both in CI [48] and in FT [42], [49], [50], there is an initial fast phase of learning that might be less specific in terms of its transfer properties, and involve higher level cognitive processes. Our rationale is to find the beginning of the second, more specific phase of learning where the initial familiarization with the task is finished, and learning mostly relies on activity and plasticity in the primary cortices. Comparison of performance levels (normalized to that of the adult performance) at the beginning of this second phase of learning in CI and FT should provide us with comparable maturational trajectories of long-range connectivity within primary visual and motor areas.

We find that there is a temporal lag between the developmental timing of primary sensory vs. motor areas; we confirm that human development is very slow in both cases, and that there is a retained capacity for practice induced plastic changes in adults.

## Materials and Methods

### Participants

Subjects were recruited from kindergartens, primary schools and universities in Budapest, Hungary. Relevant features of the subject pools in CI and FT are summarized in Table 1. Those with a history of neurological or psychiatric illness were excluded. All observers in the CI task had normal or corrected to normal vision, and those who had skeletal disorders or were professional musicians were excluded from the FT task. Written informed consent was obtained from adult subjects and the parents of participating children. Subjects were not paid for their participation. During the course of the experiment, participants were asked to report the amount of their night sleep. Those with less than 6 hours of sleep on a particular night, or those with sleep-wake cycle disruptions were also excluded from the study.

**Table 1:** Age groups of participants in the CI and FT tasks.

Age-group			CI task			FT task		
	Age (mo)		M	F	Age (mo)	M	F	R/L handed
**7 years**	89,4		5	5	84,9	6	4	9/1
**9 years**	103,6		4	6	100,8	4	5	7/2
**11 years**	132,5		5	5	132,6	5	5	9/1
**13 years**	153,6		6	4	150,5	5	5	9/1
**15 years**	176,1		5	5	173,2	4	5	7/2
**21 years**	249,6		5**30**	5**30**	246,5	5**29**	5**29**	9/1**50/8**

### Ethics statement

This study was approved by the Social Sciences Ethical review Board of the Budapest University of Technology and Economics. Written informed consent was obtained from adult subjects and the parents of participating children.

### Contour Integration Task

#### Stimuli

The contour integration paradigm was originally introduced and presented in greater detail by Kovacs & Julesz [32]. In this altered version of the task (see also [51]) images were composed of collinear chains of Gabor elements forming a horizontally positioned egg shape (target) on a background of randomly positioned and oriented Gabor patches (noise). The carrier spatial frequency of the Gabor patches was 5 c/deg and their contrast was 95%. The spacing between the contour elements was kept constant (8λ; where λ is the wavelength of the Gabor stimulus) as was the average spacing between the background elements. The signal-to-noise ratio as defined by a D parameter (D = average background spacing/contour spacing) of each image was 0.9. By keeping D at a constant level, the orientation jitter of the contour elements was varied between 0° to 24° across six difficulty levels (0°, 8°, 12°,16°, 20°, 24°, see examples in Figure 1B). A set of 40 images was presented at each of the six difficulty levels, a new shape and background were generated for each stimulus, but all of the contours had the same general size and egg-like shape.

#### Procedure

Each participant was trained in the contour integration task over five days, with an approximately twenty-four hour shift between the practice sessions. The images were presented in blocks of 10 trials, 40 stimuli at each of the six difficulty levels, in an increasing order of orientation jitter. One session lasted about 20–30 minutes. In a two-alternative forced-choice (2AFC) procedure, subjects had to indicate which direction the narrower part of the egg pointed to. Stimulus onset was 2000 milliseconds, with a fixation cross between stimuli (500 ms, or shorter if the subject responded faster). Subjects were tested binocularly, and were seated at about 0.7 m away from a 17 in. HP monitor in a normally lit testing room. Monitor resolution was set to 1280×1024. Images subtended 19.93° of visual angle vertically and 26.57° of visual angle horizontally from the testing distance. The mean luminance of the monitor was 21.5 cd/m^2^.

Psychometric functions for each subject were plotted using mean scores for each of the six levels of jitter, and threshold performance was calculated by fitting a Weibull function on the data points. Threshold was defined by orientation jitter at 75% correct performance.

### Finger-tapping Task

In the Finger-tapping task (FT) participants were asked to touch the thumb with the other fingers in a given order as quickly and precisely as possible. They were instructed not to correct errors and continue with the task without pause as smoothly as possible. Participants were asked to close their eyes, thus visual feedback was not allowed. Data acquisition started when participants were able to produce three correct sequences successively, with eyes closed. The beginning and the end of a practice block was signaled by a ‘beep’ sound from the computer The practice sequence was a four element sequence of 1-3-2-4 (1: index finger; 2: middle finger; 3: ring finger; 4: little finger). Ten blocks of 16 sequences were performed each day, with self-paced rest periods between them. The practice sessions were conducted approximately at the same time of the day through five consecutive days. On the fifth day, transfer of the practice sequence to the dominant hand (Transfer 1), and transfer to a new sequence (4-2-3-1) in both hands were also tested (Transfer 2 and Transfer 3). The three transfer tests were randomly ordered. Transfer tests are very relevant to carry out in FT in order to see whether prolonged or multisession learning involves use-dependent changes in connectivity within the neuronal populations in the primary motor cortex, in which case, lateralized motor representation results that is specific to task parameters with little or no transfer to the non-trained hemisphere or for a novel task involving the same movement elements [41]–[42], [50]. We introduced three transfer tests in order to see whether lateralized, task-specific representations have developed in M1.

A maximum motor speed task was also carried out with a new sample of participants of the same age-groups by a non-serial finger-tapping task (n = 60). In this task subjects had to touch the thumb with the index finger of the non-dominant hand as fast as possible. Blocks of 64 index finger taps were repeated three times with an at least two-minutes rest between them. Maximum motor speed was defined as the number of index finger taps/s.

#### Data acquisition

Finger-tapping data were obtained in an improved version of the original finger-tapping paradigm. Since subjects in different age-groups might have considerably varying motor abilities, we developed a data acquisition method that enables precise and automated measurement of performance without using external equipments, such as a computer keyboard. A custom-made ‘data glove,’ consisting of metal rings was placed on the participants' fingertips. Each metal ring electrode corresponded to a given finger and was connected to a laptop computer through a USB-Serial converter. The ‘data glove’ enabled participants to use their hands freely, and to close their eyes during the task. A task sequence was identified from the first element of the sequence to the next first element. For example, when a sequence of 1-3-2-4 was the task, sequences are identified and separated as follows: 1-3-2-4 – 1-3-2-4 – 1-3-2-2-4 – 1-3 – 1-3-2-4 – 1-3-2-4). Motor performance of groups with different motor abilities can only be compared by taking the speed/accuracy trade-off into account. A combined measure of speed and accuracy parameters might bring a diplomatic balance into this trade-off. Inconsistent performance also alters the length of the FT sequences, so it may vary from trial to trial, e.g., an incorrect sequence can be either two- or eight-element long. It has influence on speed and accuracy measures. Therefore, instead of using sequence based performance measures such as number of sequences in a given time, we introduced performance measures based on finger taps. In order to eliminate the speed-accuracy trade-off in the raw data, a combined index of performance rate (PR) was calculated. It is defined as the product of speed and accuracy, where speed is defined as the number of finger taps in a second (taps/s) and accuracy is defined as the ratio of the number of finger taps in correct sequences and the number of finger taps in all sequences.

When comparing perceptual and motor data, we wanted to eliminate the influence of corticospinal tract myelination level on motor speed at different ages. Corticospinal myelination level shows close correlation with maximum motor speed (see the Results section). Therefore, PR was corrected by maximum motor speed in the following way: first, we calculated FT intertap interval as 1/PR (ms); after that we subtracted the minimum intertap interval gained as 1/maximum motor speed (ms). Thus, we gained a corrected intertap interval index that is corrected both for the speed-accuracy trade-off and for the myelination effect of the corticospinal tract. These corrections led to a more precise measure of motor cortex related changes during motor learning.

### Data analysis

#### Developmental data in CI and FT

We determined perceptual and motor development based on 2nd day performance in the two tasks in order to avoid confounding cognitive effects (see the section on “Finding comparable regions in the learning curves in CI and FT” in the results section). Performance of each age-group was normalized to that of the adult performance level within each task (z score) and two-way ANOVA (learning condition×age) was performed on the records. Multiple comparisons were performed by LSD. We also conducted independent-t tests on the developmental data to compare the average performances of the age-groups.

#### Practice induced learning in CI and FT

We analyzed the learning rates in four periods (1: from Day 1 to Day 2; 2: from Day 2 to Day 3; 3: from Day 3 to Day 4; 4: from Day 4 to Day 5) in the two learning conditions. Day 1 performance was considered 100%, and performance on subsequent days was expressed relative to that. Three-way mixed ANOVA (learning condition×age×learning period) was performed on the learning data. Multiple comparisons were performed by LSD. Significance level was set at p<0.05.

## Results

**Figure 2:**
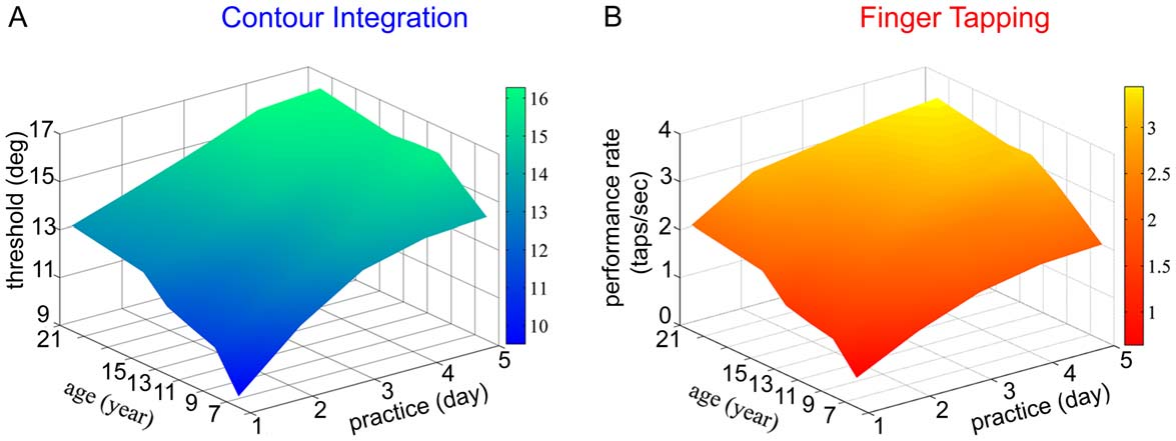
Developmental-learning surfaces. (A) Developmental-learning surface in CI. Performance threshold of each age-group is expressed in degrees of orientation jitter along the contour as a function of age and practice. Performance in CI increases as a function of age, suggesting that contour integration has a slow developmental course. Performance also increases as a function of practice, with a faster progression of learning in the younger age-groups at the beginning of practice. However, in the later phases of training, all age-groups learn at the same rate. (B) Developmental-learning surface in FT. Performance rate (number of taps/second) is expressed as a function of age and practice. Performance in FT increases both as a function of age and practice, similarly to CI.

Developmental and practice-induced learning curves are presented in the joint-spaces of Figure 2A and 2B for vision and movement, respectively. The data in Figure 2A represent the assessment of both perceptual learning capacity and developmental trajectories in CI in a sample of 60 subjects (7 to 21 years of age, 5 days of practice; see Methods). Visual CI performance increases both as a function of age (ANOVA F(5,54) = 5.41, p<0.01) and practice-days (ANOVA F(4,216) = 156.43, p<0.01). These data confirm that contour integration has a slow developmental course as it has been indicated earlier [28]. It is also confirmed that practice leads to enhanced performance levels even in adults (see also 48, 51). Although the interaction between age and practice was not significant (ANOVA F(20,216) = 1.53, p<0.1), further analysis revealed a significant main effect of age for days 1 and 2 (p<0.01), indicating that there is a faster progression of learning in the younger age-groups at the beginning of practice. However, in the later phases of training, all age-groups learn at the same rate.

Developmental and practice induced improvements of motor performance in the FT task are shown in Figure 2B (n = 58; 7 to 21 years of age, 5 days of practice; see Methods). FT performance rate (as measured in terms of the correct taps per second) increases both as a function of age (ANOVA F(5,52) = 10.76, p<0.01) and practice (ANOVA F(4,208) = 248.05, p<0.01). These results are in accordance with previous findings, where a developmental a trajectory was found in FT learning between the ages of 9 and 17 years [52]. While earlier studies indicated that the capacity to improve is preserved in adults [41], [42], the extremely slow developmental curve from childhood to adulthood in FT is reported here for the first time. We found a superior learning capacity in the younger age-groups across all 5 days of practice, as it is shown by the significant interaction between age and practice (ANOVA F(20,208) = 1.81, p<0.05).

This pattern of results indicates that both visual (CI) and motor (FT) performance improves throughout an extended developmental period in humans, and that practice induced improvements of performance are significant in all studied age-groups in both tasks. However, as indicated above, a direct comparison between the two surfaces of Figure 2 will not provide a clear view on the comparative maturational trajectories of visual and motor cortices.

**Figure 3:**
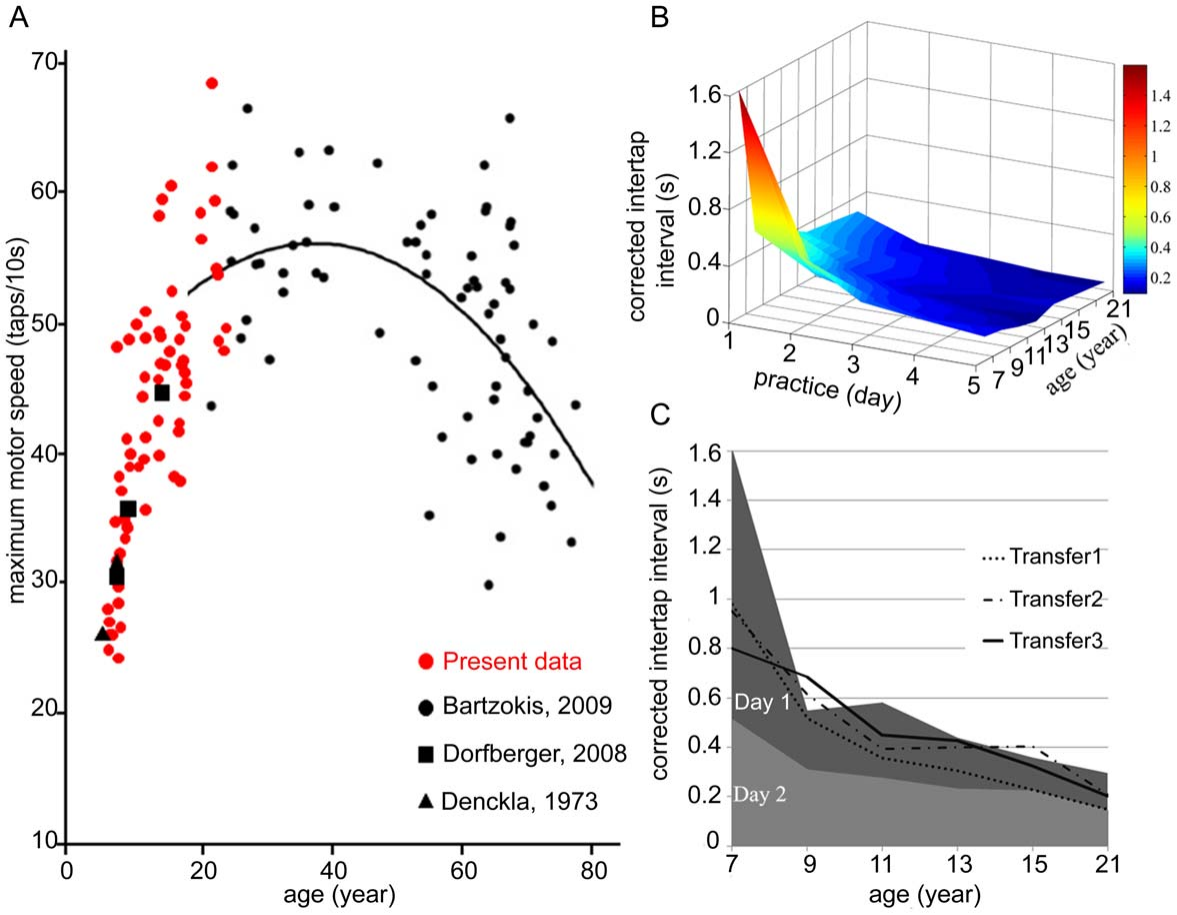
Correction of Finger-tapping data. (A) Variation of maximum finger tapping speed (FTS = finger taps/s) as a function of age. Maximum FTS is affected by corticospinal tract conduction velocity due to myelination [55] and likely has impact on developmental motor performance. (B) Developmental learning surface corrected by maximum FTS. Data are expressed as the interval between finger taps (s) in correct sequences in the serial FT task after subtraction of maximum FTS. Correction with maximum motor speed ensures that the developmental-learning surface reflects cortical plasticity with no effect of corticospinal myelinization on performance. After correction, there is a marked initial improvement at the age of 7 with no significant learning effect after the 3^rd^ day in any age-group (p<0.05). (C) Performance in transfer tests compared to Day 1 and Day 2 performance in the learning task. Transfer 1 refers to practice effects with the non-trained hand. Transfer 2 is a new task performed with the trained, and Transfer 3 with the non-trained hand. Transfer performance did not exceed Day2 performance in any of the groups (p<0.05). The lack of learning-transfer clearly indicates that processing and learning involve use-dependent changes in connectivity within the neuronal populations in the primary motor area.

As discussed in the introduction, neural correlates indicate the role of lower level visual areas in integrating the contour-in-noise stimulus a [39], [38], [40], [53], [54], in addition to its specific design that addresses the primary visual cortex. The design of the motor task allows less control over the involved cortical areas than the design of the visual task. One of the important factors affecting performance in FT is maximum finger tapping speed (FTS) that is determined by conduction velocity of the corticospinal tract due to myelination [55]. Maximum FTS shows a lifespan trajectory reaching a peak around the age of 40 years ([52], [55]–[56] see Figure 3A). Consequently, it is likely that maximum FTS has an effect on motor performance throughout the age range of the present study in a serial FT task as well. To eliminate the effect of age-related corticospinal tract conduction velocity changes, we measured FTS within the same age range as in the learning task (Figure 3A). Then we subtracted FTS from the developmental learning surface (see Methods), ensuring that such a corrected developmental-learning surface reflects cortical plasticity (Figure 3B). The role of M1 in FT was also tested by the transfer tests (the same task carried out by the non-trained hand (Transfer 1); a novel task carried out by the trained (Transfer 2) and the non-trained (Transfer 3) hand, Figure 3C). Transfer performance did not exceed Day 2 performance in any of the groups (p<0.05). The lack of learning-transfer clearly indicates that processing and learning involve use-dependent changes in connectivity within the neuronal populations in the primary motor area.

**Figure 4:**
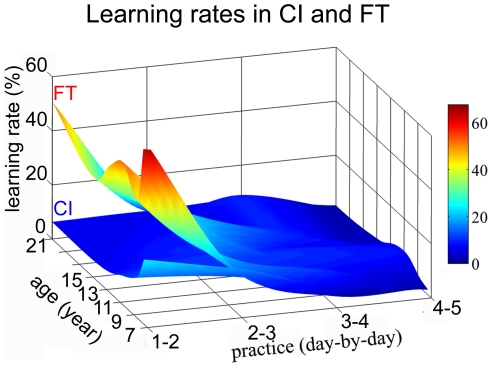
Comparison of learning rates in Contour Integration and Finger-tapping. Day 1 performance is considered 100%, and performance in subsequent days is expressed relative to that. Improvements are calculated by taking the difference between thresholds in consecutive days of practice (such as, Day 1–Day 2, Day 2–Day 3, Day 3–Day 4, Day 4–Day 5). There is a larger improvement from Day 1 to Day 2 in FT than in CI across all age-groups. This difference vanishes from Day 2 to Day 3, and learning rates become nearly equivalent in the two tasks after Day 3.

The comparability of the two tasks is a challenging issue, especially in terms of task complexity and potential cognitive load. In order to reveal differences in these, we employed learning paradigms. It has been suggested in both cases [42], [48]–[50] that the initial faster and less specific phase of learning might be related to task familiarization and higher-level cognitive processes, while in the second, slower and more specific phase, performance and improvements might be more related to primary sensory or motor cortices. In order to discern these two phases and find the second phase that would serve our perceptual and motor comparison better, here we calculate and compare session-by-session learning speed in the two tasks for all age-groups. While Figure 2 presents developmental and practice-induced learning curves in CI and FT in separate graphs, we plot learning speeds (Learning rate) within the same graph in Figure 4. As it is clearly shown in Figure 4, the two tasks are different in terms of the initial speed of learning. There is a much faster improvement from the first to the second session in FT than in CI across all age-groups (7y: t = −4,18, df = 17, p<0,01; 9y: t = −4,17, df = 17, p<0,01; 11y: t = −7,2 df = 17, p<0,01; 13y: t = −5,24, df = 17, p<0,01; 15y: t = −4,41, df = 17, p<0,01; 21,5y t = −6,06, df = 17, p<0,01). However, this large difference seems to diminish and disappear later. Improvement from the second to the third session is the same in FT and in CI, except for some relatively small differences in 9–11 year olds (9y: t = −2,29, df = 17, p<0,05; 11y: t = −2,78, df = 17, p<0,05). Learning rates become nearly equivalent in the two tasks across all ages from the third session. Different initial learning speeds can be interpreted as a difference in task complexity and/or cognitive load, while similar speeds in the later phase indicate a higher degree of comparability between task performances. Since learning rates are reasonably similar from the second day on, we propose that second day performance in CI and FT is the most advantageous for the comparison between the maturational trajectories of primary visual and motor areas using behavioral measures. Second day performance seems to satisfy both relevant conditions: (1) the second phase of learning has begun; and (2) we are still assessing maturational trajectories which are not confounded by the capacity to learn at different ages.

### Comparing Developmental trajectories of V1 and M1

In order to compare the developmental curves in FT and CI we expressed Day 2 performance of the participants in z score (Figure 1D). Performance of younger age-groups was standardized to that of the adult group. Two-way mixed ANOVA (age×learning condition) showed significant main effect for both age (F_1,5_ = 14.74, p<0.01) and learning condition (F_5,108_ = 30.45, p<0.01) with significant age×learning condition interaction (F_5,108_ = 6.13, p<0.05). We found significant differences between CI and FT performance at age 7 (CI z-score = −1,264, FT z-score = −5,2852, t = −5,150, df = 18, p<0.01), at age 9 (CI z-score = −0.767, FT z-score = −2,360, t = −2,3515, df = 18, p<0.05) and at age 15 (CI z-score = −0.2998 FT z-score = −0.9728, t = −2.09, df = 17, p = 0.052). In order to see whether there is a difference in the performance of adults and 15-year-old children, we employed an independent t-test. There was no significant difference in CI (t = −0,775, df = 18, p = 0,449), however 15-year-old children performed significantly below the adult level in FT (t = −2,415, df = 17, p = 0,027). These results imply that fine motor functions are not operating at the adult level in terms of speed and accuracy at the age of 15, while contour integration reaches the adult level at this age. Since CI and FT both address long-range connectivity in primary visual and primary motor cortices, respectively, we suggest that the functional development of long-range lateral intralaminar connections in humans is slower in the primary motor cortex than in the primary visual cortex.

## Discussion

We employed behavioral paradigms, a Contour Integration test and a Finger-tapping task, to assess the functional maturity of long range horizontal cortico-cortical connections in primary visual and primary motor areas. Several earlier studies revealed that these tasks require long-range integration within the primary cortices. In addition to applying these well-established methods, we carefully eliminated possible confounding factors, such as different task requirements (complexity, cognitive load) by using these tasks in a learning paradigm. We have shown that initial performance levels might not be appropriate for comparisons since the rate of performance improvement is significantly different from the first to the second practice session (Day 1 to Day 2) across tasks and across age-groups. However, this first, and highly variable phase of learning, probably involving higher level cognitive processes, seems to be over by the second session (Day 2), and performance improvement proceeds at the same rate in both tasks and all age-groups. Therefore, it appeared reasonable to use Day 2 data in deriving and comparing the two developmental curves. In the case of the Finger-tapping task, the impact of myelination and age-related changes in corticospinal tract conduction had to be considered as well. To this end, we registered the maximal speed in a single finger-tapping task (determined mainly by corticospinal tract conduction velocity) in each age-group, and deduced it from the sequential finger-tapping data. The resulting values are believed to reflect cortical network functioning.

Following the above mentioned corrections, our results show that the developmental curves in the perceptual (CI) and in the motor (FT) tasks are not overlapping. Although both curves are demonstrating protracted development, extending well into the teenage years, motor development, as measured by the FT task, is relatively more delayed: fine motor coordination is not reaching adult levels in terms of speed and accuracy by age 15, while perceptual integration is adult like at this age.

Greater capacity to cortical plasticity in M1 may stem from the more distributed organization of M1. While M1 consists of distinct representations of larger body parts (e.g., the hands), within these functional subregions, a widely distributed and overlapping representation system exists, involving horizontal connections [46]. It has been suggested that such an organization is more advantageous to provide greater capacity for storage and to contribute to flexibility [17], [46]. Flexibility is crucial in generating a wide repertoire of movements, including ones not performed previously. Maintaining this repertoire requires the ability to have access to a large number of combinations of muscle contractions. Similarly, during the acquisition of new skills this aforementioned distributed type of network in M1 could be reorganized to represent new combinations more rapidly, while a discrete somatotopic representation would limit this capacity [45]–[46]. The extremely extended temporal window, during which experience can shape the fine functional connections, might be explained by the fact that the size of various body parts and the proportion of body parts are exposed to enormous alterations. Furthermore, daily motor performance in our continuously changing physical environment puts a permanent constraint on the motor system. To adjust to these constraints, the system has to continuously create novel movements. The prolonged time course of the maturation of the primary motor connections might be necessary to maintain a higher capacity of the system to meet these requirements mentioned above.

Our behavioral data, suggesting that the functional maturation of long-range lateral intralaminar connections and the refinement of these neocortical networks in primary motor cortex are slower than that of the primary visual cortex in humans, are in line with histological (e.g. pruning or GABAergic network properties [57]–[58]), and psychophysiological (e.g. synchronized oscillations [59]) accounts indicating that changes incidental to development occur earlier in the primary visual than in the primary motor region. Studies of developing horizontal connections often emphasize that collateral pruning and selective synapse elimination are important for achieving functional maturity (e.g. [60]). Synapse production continues postnatally, and after an initial overproduction, synaptic density reaches its peak in infancy [61]. Following this peak, there is a prolonged selective elimination of the connections, resulting in a structural and functional alteration in neuronal circuits. Synaptic density decreases to adult values during late childhood and early adolescence, however, synaptic elimination and network refinement occurs in a hierarchical pattern in the human cortex: primary sensory areas develop first, followed by the maturation of the motor and association cortices, while the prefrontal cortex develops last [57]. Synaptic density in V1 decreases to adult levels by 10 years of age [57]. With respect to M1, synaptic density remains elevated until the age of 10 and decreases to adult values in late childhood and early adolescence [62].

The development and maturation of cortical networks strongly depends on neuronal activity, whereby synchronized oscillations play an important role in the stabilization and pruning of connections. There are significant oscillations during childhood and adolescence, e.g. there is a reduction in the amplitude of oscillations that is predominantly pronounced for delta and theta activity [63]. This developmental change occurs more rapidly in posterior than in frontal regions [59], and takes place earlier in the primary visual than in the primary motor area.

In addition to the number of connections, the types of connections are equally important in the functioning of cortical networks. An appropriate balance between excitatory and inhibitory synaptic inputs appears to be necessary. GABAergic interneurons play a pivotal role in establishing neural synchrony in local circuits. It was demonstrated that a single GABAergic neuron might be sufficient to synchronize the firing of a large population of pyramidal neurons [64]. In the human visual cortex, studies on the developmental changes in GABAergic mechanisms in postmortem tissues have shown that the relevant changes start to occur between the ages of 10 and 13 years of age [58]. Although there are no postmortem studies on GABAergic mechanisms in the motor cortex, it has been shown that both N-methyl-Daspartate receptor activation and GABAergic inhibition play a crucial role in use-dependent plasticity in the human motor cortex [65]. Furthermore, in a TMS study it was confirmed that the GABAergic interneuron system does not function at an adult level even in adolescence in the motor cortex [66].

In conclusion, we confirm that human development is very slow both in the primary visual and motor domains, and we find a retained capacity for practice induced plastic changes in adults. Based on the temporal lag between the developmental timing of primary sensory vs. motor functions, we suggest that the ontogenetic maturational rate of the intracortical horizontal connections in the primary motor cortex is slower than that of the primary visual cortex, providing a wider temporal window for experience-dependent plasticity in the motor system. Our results seem to be in strong correlation with anatomical and physiological data on the developmental order of different cortical areas. This pattern of results also raises the possibility of human-specific development of the “canonical circuits” of primary sensory and motor cortices, perhaps reflecting the ecological requirements of human life.
